# Five new species of *Syzygium* (Myrtaceae) from Sulawesi, Indonesia

**DOI:** 10.3897/phytokeys.81.13488

**Published:** 2017-06-15

**Authors:** Fabian Brambach, James W. Byng, Heike Culmsee

**Affiliations:** 1 Plant Ecology and Ecosystems Research, Albrecht-von-Haller Institute for Plant Sciences, University of Göttingen, Untere Karspüle 2, 37073 Göttingen, Germany; 2 Plant Gateway, 5 Talbot Street, Hertford, Hertfordshire, SG13 7BX, UK; 3 Naturalis Biodiversity Center, Botany, P.O. Box 9517, 2300 RA, Leiden, The Netherlands; 4 DBU Natural Heritage, German Federal Foundation for the Environment, An der Bornau 2, 49090 Osnabrück, Germany

**Keywords:** Indonesia, Lore Lindu National Park, Myrtaceae, Sulawesi, *Syzygieae*, *Syzygium*, taxonomy, Wallacea

## Abstract

Following ongoing ecological research on the tree diversity of the Indonesian island of Sulawesi, we describe five new species of *Syzygium*. These are the first descriptions of *Syzygium* species from the island since Blume (1850, *Jambosa celebica* and *J. cornifolia*), highlighting the significant lack of taxonomic research on the genus for the region. The five species proposed as new are *Syzygium balgooyi* **sp. nov.**, *Syzygium contiguum* **sp. nov.**, *Syzygium devogelii* **sp. nov.**, *Syzygium eymae* **sp. nov.**, and *Syzygium galanthum* **sp. nov.** All species are illustrated and information on their distribution, ecology, and conservation status is given.

## Introduction

The botanical diversity of the Indonesian island of Sulawesi is poorly known and remains one of the least studied in Southeast Asia (de Vogel 1989, Cannon et al. 2007). The "Checklist of woody plants of Sulawesi" by Kessler et al. (2002), the most comprehensive taxonomic work for the island, highlighted how numerous taxonomic groups were in need of specialist systematic work. Myrtaceae and the largest genus in the family, *Syzygium* P.Browne ex Gaertner (1788, 166), were in particular emphasised because only four species of *Syzygium* were recorded in the checklist while approximately 350 un-named collections were listed. Several species now accepted as belonging to *Syzygium* were listed under other generic names in the checklist, such as *Acmena* de Candolle (1828, 262) and *Eugenia* Linnaeus (1753, 470), which further underscores the complex history of the genus.

*Syzygium* is the most - species rich genus of woody plants in Southeast Asia with around 1000 species but little is known of the genus in Wallacea, the biogeographically important transition zone between the Asian and Australian continental areas. As in the other Wallacean regions, the Maluku Islands and Lesser Sunda Islands, the *Syzygium* species of Sulawesi have never been revised or monographed so there is no robust baseline data of which species occur in the region. The last *Syzygium* species to be described from Sulawesi were by Blume (1850) under the generic name *Jambosa*: *J. celebica* Blume and *J. cornifolia* Blume. The occurrence of other, mostly widespread species of *Syzygium*, have been noted over time and resulted in 14 species recorded from Sulawesi at present (WCSP 2016). This number is unrealistically low when considering *Syzygium* diversity in neighbouring regions: Java holds c. 60 species; Borneo, the Philippines, and New Guinea c. 200 species each (WCSP 2016). In fact, recent extensive examination of herbarium material suggests that Sulawesi harbours > 100 species, the great majority of them yet unnamed (SYZWG 2016).

Species of *Syzygium* are present in virtually all ecosystems of Sulawesi, and are often important components of the biological communities (van Balgooy and Tantra 1986, Whitten et al. 1987, Milliken and Proctor 1999, Culmsee et al. 2010), so the lack of taxonomic resolution presents a serious impediment for a better understanding of ecological processes as well as for conservation efforts on the island.

In 2006–2007 and 2011–2012, the University of Göttingen, Germany, and Tadulako University, Palu, Indonesia conducted ecological fieldwork campaigns in Lore Lindu National Park (LLNP), Central Sulawesi. Difficulties in the identification of *Syzygium* specimens collected during these surveys motivated us to take a closer look at the taxonomy of the genus. Fortunately, the area of LLNP had been visited before by other botanists (Bloembergen 1940, Meijer 1983, van Balgooy and Tantra 1986), so good fertile collections for comparison were available in herbaria. Five species could not be matched with previously published taxa and are here proposed as new. This is the first time in > 165 years that species of *Syzygium* are described from Sulawesi.

## Methods

### Morphological observations

The specimens collected during our ecological fieldwork in LLNP (HC, 2006–2007; HC and FB, 2011–2012) were the starting point for this study. Duplicates of relevant specimens, including types, were deposited in L and the Indonesian herbaria BO and CEB (herbarium acronyms follow Thiers continuously updated). To identify our specimens, all *Syzygium* specimens from Sulawesi at A, B, BM, BO, E, GH, K, L, M and U were examined and all matching specimens sorted into morphospecies. We then attempted to identify our morphospecies using keys and floristic treatments from regions around Sulawesi: the Malay Peninsula (Henderson 1949), Borneo (Merrill and Perry 1939, Ashton 2011), Java (Amshoff 1963), the Philippines (Robinson 1909, Elmer 1914, Merrill 1915, 1921, 1951, Pelser et al. 2011), New Guinea (Diels 1922, 1924, Merrill and Perry 1942, Hartley and Perry 1973), and Australia (Hyland 1983). Because *Syzygium* is such a species-rich genus, we had to repeat this process several times to make sure we did not miss any species our specimens could be matched with. All specimens examined by us are marked with an exclamation mark. We recorded morphological characters of all cited specimens to produce the species descriptions using the package *monographaR* (Reginato 2016) in R (R Core Team 2015).

Photographs in the field were taken using a Canon EOS 500D camera with a Tamron AF 18–200mm f/6.2–38 lens, for later photographs of dried material we used the same camera with a Tamron SP 90mm F/2.8 MACRO lens. Colours of dried specimens were compared to Munsell Soil-Color Charts (Munsell Color 2010) and colour names used accordingly.

Wood density (oven-dry mass per fresh volume) was determined from wood cores extracted with increment borers. The samples’ fresh volume was measured by Archimedes’ principle and weight was noted from the same samples after oven-drying for 48h at 105°C.

For the descriptions, flowers and fruits were boiled in dilute detergent for 5 minutes and dissected thereafter. Dimensions were measured using a ruler with 0.5 mm accuracy. All colours and measures given refer to dried and pressed material unless otherwise stated. We measured the distance of intramarginal veins from leaf margin in the proximal 2/3 of the blade; it usually decreases towards the apex. Likewise, we measured the distance of secondary veins in the central ½ of the leaf; it decreases near the base. Dimensions of flower buds are given including the anthopodium, those of the hypanthium excluding the anthopodium (if present).

### Terminology

Terminology for organs in *Syzygium* has been varied and often confusing, with authors using different terms for the same structures or similar terms for different structures.

We here adopt the detailed concepts of Briggs and Johnson (1979) on inflorescence structure but use more common terms instead of their rather technical vocabulary: bract instead of *pherophyll* and bracteole instead of *prophyll*/*metaxyphyll*. We follow Briggs and Johnson (1979) in using the term *anthopodium* for the internode between the flower and its subtending bracteoles. This structure has been referred to as pseudopedicel (Schmid 1972) or pseudostalk (Henderson 1949), but Briggs and Johnson (1979) convincingly argued that it is indeed the last internode below the flower and coined the term anthopodium to avoid further confusion. The concept has been adopted by Hyland (1983) who, however, used the more common term pedicel. The anthopodium may be elongated or not; in the latter case the flowers are sessile, although they may appear stalked when arising from elongated higher-order axes of the inflorescence. Otherwise we follow the terminology of Beentje (2012) and Hyland (1983) except for using *hypanthium* instead of *calyx tube*.

### Presentation of data

Since several specimens found in herbaria contained very limited information about the respective collecting localities, we interpreted the locality data of all specimens cited in this paper and translated it into a common format. The format contains approximate coordinates in WGS 84 (if not given on the label), the nearest village, and the administrative divisions in descending order: Province, Kabupaten (Kab., Regency), and Kecamatan (Kec., District).

Specimens collected in Sulawesi by the Forest Research Institute Buitenzorg (Bogor), also called Boschproefstation or Boschbouwproefstation (van Steenis-Kruseman and van Welzen 2014), often bear confusing information about the respective collectors. The original herbarium labels for these collections (usually deposited in BO) give the actual collector with a personal collection number and in addition the institutional *bb*- or *Cel.*-number. Duplicate labels usually only contain the institutional number and either read Neth. Ind. For. Service or Boschproef station as collector. We cite these specimens as NIFS (Netherlands’ Indies Forest Service) with the respective institutional number.

In the diagnoses, we give floral formulas for each species, following the format and recommendations of Prenner et al. (2010). Furthermore, we apply Appropriate Citation of Taxonomy (Seifert et al. 2008) throughout the manuscript.

Under Distribution and Habitat, we characterised the forest stands of the species which were found primarily in our (FB and HC) inventory plots by mentioning the families with the five highest family importance values (FIV). The FIV is an objective measure of importance of a family in a stand taking into account the number of individuals, number of species, and basal area of that family and comparing them to the stand total (see Mori et al. 1983 for detailed description of the method).

### Conservation Assessment

We used GeoCAT (Bachman et al. 2011) to calculate the extent of occurrence (EOO) and area of occupancy (AOO) of each species as basis for the conservation assessments following the recommendations of IUCN Standards and Petitions Subcommittee (2014).

## Results

All species here described are glabrous in all parts and possess flower characters placing them in the broadly defined Syzygium subg. Syzygium (Craven and Biffin 2010): anther sacs parallel and opening by longitudinal slits, placentation axile-median. The species of which we have seen fruiting material furthermore have seeds without intrusive tissue interlocking the cotyledons and free cotyledons, conforming with subgenus Syzygium as well. These characters are not mentioned again in the species descriptions.

### 1. Syzygium balgooyi

Taxon classificationPlantaeMyrtalesMyrtaceae

Brambach, Byng & Culmsee sp. nov.

urn:lsid:ipni.org:names:60474721-2

Figures 1 , 2 , 8

Eugenia “spec. BB“ (Koorders 1898, 173, 459, Koorders-Schumacher 1914, 95).
Myrtaceae „ sp. 10“ p.p. (Culmsee and Pitopang 2009, see also 2017 (Erratum), Culmsee et al. 2011).

#### Diagnosis.

*Syzygium balgooyi* is characterised by long, elongate-clavate flowers, a character otherwise only known from the morphologically similar *Syzygium schumannianum* (Nied.) Diels (1922, 402) from New Guinea and the Maluku Islands. *Syzygium balgooyi* differs from that species by its smooth (vs prominently longitudinally ridged) hypanthium and fruit and by the hypanthium rim which remains entire after anthesis (vs apically splitting into 4 recurving lobes). Floral formula B1 Bt2 K4* C4* A∞* Ĝ(2)┼ Vx∞.

#### Type.

INDONESIA. South Sulawesi (Sulawesi Selatan), Kab. Luwu Timur, Kec. Nuha, Between Soroako and Nickel plant site, c. 2°33'S, 121°22'E, 500 m, 10 Jul 1979: *van Balgooy 3956* (flowers; holotype L [L.2517558]! [spirit collection L 0771145] [wood sample L 0708624], isotype A [A01143212]!).

#### Description.

**Trees**, up to 37 m tall, diameter at breast height ≤ 65 cm, trunk straight, ≤ 20 m tall, often fluted and with buttresses ≤ 3 m tall and 1 m out. Outer **bark** pale brown to bright red, peeling off in small or large sheets, inner bark dark red, usually paler towards inside, sometimes with little watery red sap, wood very hard and heavy, sapwood cream, clearly separated from the dark reddish brown heartwood. Young **branchlets** 1–2 × 1.5–4 mm, strongly flattened, the flat sides usually with two lateral, rounded ridges leading to the petioles and one central ridge continuing into the next internode, often resinous when dry, epidermis green, drying dusky red to reddish black and usually smooth; becoming terete, bark drying red to dark reddish brown, finely flaking and with conspicuous flaking remnants of epidermis.

**Leaves** (sub-)opposite. Petioles 2–12 × 1–3.5 mm, flat and sometimes narrowly winged above, rounded or keeled beneath, drying reddish black and smooth. Blades (4–) 7–11.5 (–16) × (1.5–) 3–5 (–9) cm, ratio (1.2–) 1.8–2.7 (–5), (narrowly) elliptic, obovate, or oblanceolate, base cuneate and attenuate at the very base or obtuse to rounded, apex usually rounded or obtuse, sometimes emarginate or acute, margin slightly to strongly revolute; (thick-)coriaceous, purple, pink, or reddish when young, fresh to dark glossy green above, paler glossy green beneath, drying dull to shiny, often resinous after drying, reddish brown to reddish black above, reddish brown to very dusky red beneath. Midrib channelled above, prominent and rounded or keeled, drying reddish black and smooth beneath. Secondary vein pairs (9–) 11–14 (–16), 4–12 (–15) mm apart, ± faint and lighter red than the lamina above, ± prominent and darker than the lamina beneath; intersecondary veins present. Tertiary veins sup-parallel near the midrib, reticulate towards the margins, ± faint above, faint or prominulous and darker than the lamina beneath. Inner intramarginal vein 1–5 mm from the leaf margin, ± looping; outer intramarginal vein < 1 mm from the leaf margin, often seemingly absent from leaf margin.

**Inflorescences** terminal and often in axils of distal leaf pair, rather dense panicles, 5–10 cm long, peduncles 1–6 cm long, axes subangular or rounded, flattened, resinous after drying. Bracts c. 3 mm long, linear, pellucid-dotted, caducous; bracteoles 2 per flower, sometimes seemingly 4 (by contraction of the ultimate inflorescence axes?), 1 mm long.

**Flowers** 5–15 per inflorescence, within the panicles in monads or clusters of 2–4, 4-merous, anthopodium absent, c. 20–30 mm in diameter at anthesis, mature buds 20–30 × 3–6 mm. Hypanthium 20–30 × 5–7 mm, elongate-clavate, yellowish green, drying smooth black, hypanthium rim 15 mm long, glandular inside. Calyx lobes c. 2 × 2 mm, claw- or hood-shaped. Petals c. 4 × 3 mm, ± obovate, pale green. Stamens c. 100, filaments 10–20 mm long, pale green, anthers c. 0.5–0.8 mm long, ellipsoid, yellow. Ovary bilocular, locules surrounded by spongy tissue, ovules c. 15–20 per locule, ascending, ± arranged in 2 longitudinal rows. Style 25–35 mm long, pointed.

**Fruits** 1–2-seeded, 27–33 × 12–16 mm, ampulliform, yellowish green (immature?), drying black, smooth or slightly warty, pericarp c. 1 mm thick, leathery when fresh, ± woody when dried, hypanthium rim 8–12 mm long, 4–5 mm in diameter.

**Seeds** 13–15 × 9–10 mm, ellipsoid, testa cartilaginous, attached to the pericarp, cotyledons free from the testa, ± half-globose, minutely verrucose, facing surfaces undulate.

#### Etymology.

The species is named after Max Michael Josephus van Balgooy (*1932), botanist and authority on Southeast Asian plant taxonomy, identification, and biogeography. He collected over 900 specimens during a Dutch-Indonesian expedition to Sulawesi in 1979, among them the type specimen of this species. We enjoyed the privilege of learning from Max during several stays at the herbarium in Leiden and receiving his help with the identification of our specimens collected in Central Sulawesi.

#### Phenology.

Flowering specimens have been encountered throughout the year without any apparent association with geography or climate. Fruiting specimens have been recorded in May (*de Vogel 5413*) and September (sight record by FB).

#### Distribution and habitat.

*Syzygium balgooyi* is restricted to Sulawesi and widespread across the island (Figure 2). The species occurs on a variety of geological substrates, namely volcanic rocks on the Northern Peninsula, acid plutonic rocks and schists in the Central Sulawesi Mountains (see Brambach et al. 2016 for definition), alluvial deposits at the base of the Southern Peninsula, and ultramafic rocks on the Eastern and Southeastern Peninsulas. According to the information on specimen labels it grows in primary forests, both virgin and disturbed, over a wide elevational range (c. 100–2000 m). There, it forms part of the canopy layer, sometimes co-dominant (van Balgooy and Tantra 1986), but usually with scattered individuals (Culmsee et al. 2011, Brambach et al. in press).

#### Conservation status.

The AOO of 64 km² would place *Syzygium balgooyi* in the category “Endangered” (EN), despite its wide distribution in Sulawesi (Figure 2) as reflected by the estimated EOO of 94 451 km². The species has been found in a wide variety of habitats, including montane forests at different elevations, with scattered individuals or even co-dominant at times (see Distribution and Habitat above). We have no reason to believe that it is scarce throughout its range. Rather, we argue that the small estimated AOO is an artifact due to the generally low collection rate in Sulawesi and the real geographic distribution does not meet criterion B for any of the “threatened” categories of IUCN (2012). However, although we lack real evidence about possible changes in population size over time, using the Global forest change website (Hansen et al. 2013), we detected deforestation activities at or near five of the 18 collection localities (28%) of *S. balgooyi*. Given that the species is only recorded from old-growth forest habitats, we consider this a loss of suitable habitat, slightly below the 30% threshold for the “Vulnerable” category. Notably, all deforestation took place in places with relatively easy access and at low elevations. Thus, given (1) the relatively large EOO of *S. balgooyi*, (2) its apparent wide ecological niche, (3) its frequency of occurrence, (3) the low collection rates in Sulawesi, and (4) the loss of suitable habitat, we propose a preliminary extinction risk assessment of “Near Threatened” (NT) following the IUCN Red List Categories and Criteria (IUCN 2012).

#### Vernacular names.

Cenke hutan (= forest clove, Indonesian, *de Vogel 2651*), Jambu (general name for *Syzygium*, Indonesian, *NIFS bb 33081*), Rokobako (*NIFS Cel./II-385*), Tambeanitu (Bahasa Behoa, *Brambach et al. 1047, 1083, 1290, 1316*), Wawahuling (Bahasa Tondano, *Koorders 182*51, see Koorders 1898, 173, 459).

#### Notes.

**Figure 1.**
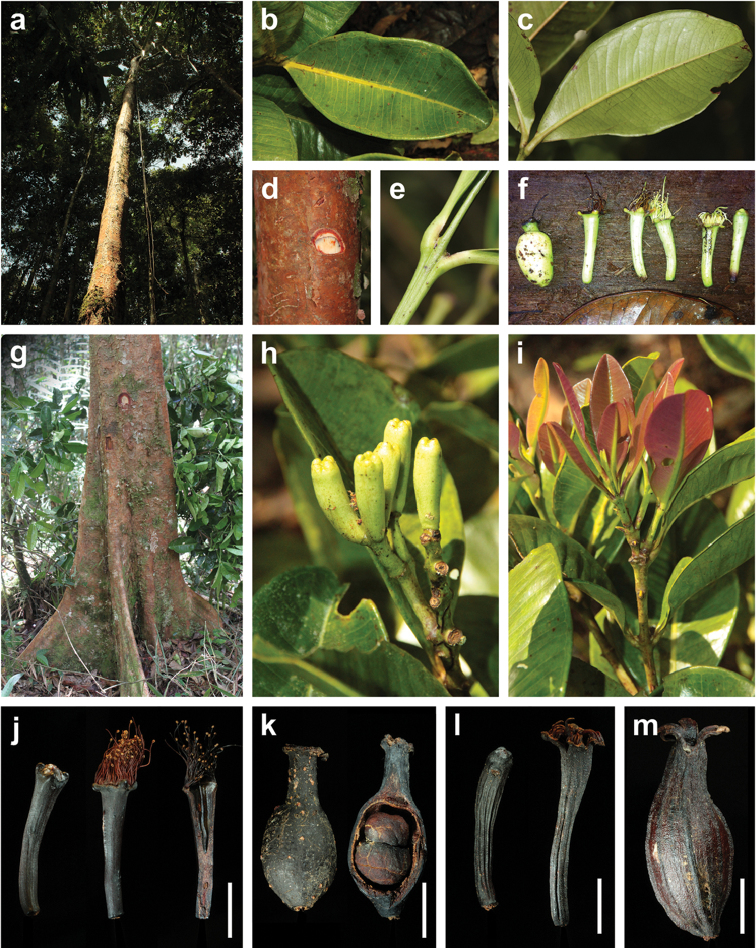
Morphological characters of *Syzygium balgooyi* and *S. schumannianum*. *Syzygium balgooyi* (**a–k**): **a** c. 20 m tall trunk **b** upper leaf surface; **c** lower leaf surface **d** bark with slash **e** ridged shoot apex with subopposite leaves **f** flowers at different stages during anthesis and fruit **g** trunk base with steep narrow buttresses **h** inflorescence with flower buds **i** shoot with young leaves **j** dried flowers before and during anthesis and longitudinal section of flower **k** dried fruit and longitudinal section of fruit showing two cotyledons. *Syzygium schumannianum* (**l–m**): **l** dried flowers before and during anthesis **m** dried fruit. **a–b** and **h–i** *Brambach et al. 1564* **c** *Brambach et al. 0861* **d–e** *Brambach et al. 0628* **f** sighting on Mt Katopas by FB **g** *Brambach et al. 0889*; **j** holotype *van Balgooy 3956* [L.2517558] **k** *de Vogel 5413* [L.2517563] **l** *Wiakabu et al. LAE 50571* [L.2535534] **m** *Brass 13610* [L.2524420]. All scale bars: 1 cm.

Among *Syzygium* species of Sulawesi, *S. balgooyi* can be recognised in the field by its tall stature (Figure 1a), the bright red bark that peels off in thin sheets (Figure 1d, g) and the rather thick, usually obovate or oblanceolate leaves with ± rounded tips (Figure 1b–c, 8a–e). Dry specimens are recognisable by the dark reddish brown twigs bearing thick black flakes of the peeling epidermis and the very dark upper leaf surface with contrasting paler veins.

Leaf size and thickness are quite variable (Figure 8a–e), as can be expected for a species with such a wide ecological distribution. Small leaves are usually found at higher elevations, whereas thick leaves seem to be associated with ultramafic soils. While the extreme forms suggest that several distinct species are involved, when taking into account all the available material, intermediate states connecting the extremes appear. We therefore prefer to treat this as one species with the vegetative parts morphologically variable.

*Syzygium balgooyi* and *S. schumannianum* are difficult to separate in vegetative state. *S. balgooyi* usually has leaves with rounded, obtuse, emarginate, or acute tips, whereas they are shortly acuminate in *S. schumannianum*, but there are exceptions in both species. Flowers and fruits of the two species also share the same structure but there are two important differences which we consider sufficient to warrant specific separation: Firstly, as indicated by the original name *Eugenia neurocalyx* Schumann nom. illeg. (in Schumann and Hollrung 1889, 90), the outer surface of the hypanthium in *S. schumannianum* bears prominent “nerves”, i.e. longitudinal ridges (Figure 1l–m). These ridges are already visible in young flower buds and remain present until the fruiting stage. Single, very faint ridges may appear in flowering specimens of *S. balgooyi* (seen in *de Vogel 2651*) but in the bulk of the material at our disposition, flower buds, flowers, and fruits are completely smooth (Figure 1j–k). Furthermore, in *S. balgooyi* the stamens are arranged in a ring along the upper margin of the hypanthium rim, which remains entire through the fruiting stage (Figure 1j–k). In *S. schumannianum*, the apical portion of the woody hypanthium rim splits into 4 outward-curving lobes and the stamens are arranged in a small area at the inside of each lobe near its tip (Figure 1l–m, Schumann and Hollrung 1889, Merrill and Perry 1942).

The wood of *S. balgooyi* is used for construction in North Sulawesi, but is not water-resistant (Koorders 1898, 173). Several collectors describe it as very hard and heavy. Mean wood density, as measured from 13 wood cores in LLNP was 0.74 g cm^-1^ (± 0.05 SD).

#### Additional specimens examined

(Paratypes). INDONESIA. **North Sulawesi (Sulawesi Utara)**: Kab. Minahasa, Kec. Kakas, Old-growth forest Pinamorongan, c. 1°08'N, 124°56'E (“Noord-Celebes, Residentie Menado, Pinamorongangebergte bij Kakas”), 500 m, 30 Jan 1895: *Koorders 18251* (sterile; L [L.2517502]! [L.2535743]!).

Kota Bitung, Kec. Ranuwulu, southern part of Wiau Forest Reserve (Hutan Lindung G. Wiau), base of Mt Klabat, c. 1°28'N, 125°03'E, 400 m, 1 Nov 1973: *de Vogel EF 2651* (flowers; L [L.2535729]! [L.2535730]! [wood sample L 0204047]).

**Central Sulawesi (Sulawesi Tengah)**, LLNP: Kab. Poso, Kec. Lore Utara, west slope of Mt Rorekautimbu, c. 1°16'S, 120°16'E, 1700 m, 15 May 1979: *van Balgooy MMJ 3371* (sterile; L [L.2535697]!).

Kab. Poso, Kec. Lore Utara, west slope of Mt Rorekautimbu, c. 1°16'S, 120°17'E, 2000 m: 5 May 1979: *Tantra IJM 1589* (sterile; L [L.2517457]!), & *1592* (sterile; L [L.2535672]!); ibid. loco, 17 May 1979: *de Vogel EF 5413* (fruits; BO [BO-1686561], K [K001024419]!, L [L.2517562]! [L.2517563]!, [wood sample L 0708565]).

Kab. Poso, Kec. Lore Utara, 4 km E of Wuasa, c. 200 m N of Rumuku waterfall, tree-inventory plot Torongkilo, 1°24.9'S, 120°16.7'E, 1450 m, 6 Mar 2012: *Brambach F, Mangopo H, Firdaus, Faber M, Tiranda R 1478* (sterile; BO [BO-1938440]!, CEB, L!) & *1564* (flower buds; BO [BO-1938441]!, CEB, K [K000993483]!) & *1583* (sterile; GOET [GOET020022]!).

Kab. Poso, Kec. Lore Tengah, 9 km NW of Bariri, 100 m E of climate tower, tree-inventory plot Bariri NE, 1°39.4'S, 120°10.5'E, 1400 m: Jul 2007: *Culmsee H y896* (sterile; CEB, L!); ibid. loco, 21 Aug 2011: *Brambach F, Mangopo H, Firdaus, Faber M, Tiranda R 0861* (sterile; BO [BO-1938438]!, CEB, GOET [GOET020025]!) & *0889* (sterile; BO [BO-1938439]!, CEB, L!) & *0907* (sterile; CEB, GOET [GOET020024]!, L!).

Kab. Poso, Kec. Lore Tengah, 9 km NW of Bariri, 80 m south of climate tower, tree-inventory plot Bariri S, 1°39.5'S, 120°10.4'E, 1400 m, Jul 2007: *Culmsee H 1459* (sterile; CEB, GOET [GOET020006]!) & *1495* (sterile; BO [BO-1938457]!, CEB); ibid loco, Jul 2007: *Culmsee H r808* (sterile; CEB, GOET [GOET020008]!).

Kab. Poso, Kec. Lore Tengah, 7 km WNW of Hanggira, E flank of Mt Dali, tree-inventory plot Pantakleabae, 1°42.0'S, 120°09.0'E, 1950 m: 3 Mar 2011: *Culmsee H, Brambach F, Mangopo H, Firdaus, Faber M, Tiranda R r2162* (sterile; CEB, GOET [GOET020021]!) & *r2254* (sterile; BO [BO-1927087], CEB, GOET [GOET020023]!); ibid. loco, 30 Mar 2011: *Brambach F, Mangopo H, Firdaus, Faber M, Tiranda R 0038* (sterile; BO [BO-1926965], CEB, GOET [GOET020027]!, K [K000993482]!, L!) & *0058* (sterile; BO [BO-1926969]!, [BO-1926970]!, CEB, GOET [GOET020033]!) & *0082* (sterile; BO [BO-1938382]!, CEB, GOET [GOET020030]!, L!) & *0097* (sterile; CEB, GOET [GOET020029]!, L!); ibid. loco., 23 Jan 2012: *Brambach F, Mangopo H, Firdaus, Faber M, Tiranda R 1333* (sterile; CEB, GOET [GOET020020]!, L!).

Kab. Sigi, Kec. Kulawi, 2.4 km ENE of Toro, NE edge of Pono Valley, tree-inventory plot Pono, 1°29.7'S, 120°03.4'E, 1050 m: 4 Aug 2006: *Culmsee 125* (sterile; BO [BO-1938456]!, CEB, L!) & *209* (sterile; CEB, K [K000993486]!); ibid. loco, Jul 2007: *Culmsee r211* (sterile; CEB, GOET [GOET020009]!).

Kab. Sigi, Kec. Kulawi Selatan, 4 km E of Watukilo, following footpath to Mt Tokepangana, tree-inventory plot Tokepangana, 1°36.9'S, 120°04.4'E, 850 m, 16 Apr 2011: *Brambach F, Mangopo H, Firdaus, Faber M, Tiranda R 0176* (sterile; BO [BO-1926967]!, CEB, GOET [GOET020028]!, L!) & *0206* (sterile; BO [BO-1926968]!, CEB) & *0283* (sterile; BO [BO-1926973]! [BO-1926974]!, CEB, GOET [GOET020032]!, K [K000993481]!, L!) & *0319* (BO [BO-1926934]!, CEB) & *0332* (BO [BO-1926966]!, CEB) & *0363* (BO [BO-1926919]!, CEB).

Kab. Sigi, Kec. Kulawi Selatan, 4 km ENE of Watukilo, 400 m N of Mboe River, tree-inventory plot Rantena, 1°36.2'S, 120°04.5'E, 700 m: 17 Jun 2011: *Brambach F, Mangopo H, Firdaus, Faber M, Tiranda R 0466* (sterile; BO [BO-1938383]!, CEB, GOET [GOET020031]!); ibid. loco, 21 Jun 2011: *Brambach F, Mangopo H, Firdaus, Faber M, Tiranda R 0628* (sterile; CEB, GOET [GOET020026]!, L!).

Kab. Sigi, Kec. Nokilalaki, 4.3 km SSW of Tongoa, NW flank of Mt Nokilalaki, ca. 400 m S of Shelter 2, tree-inventory plot Nokilalaki 2, 1°14.6'S, 120°09.1'E, 1850 m, Sep 2007: *Culmsee 2923* (sterile; CEB, L) & *3075* (sterile; BO [BO-1938463]!, CEB).

Kab. Sigi, Kec. Nokilalaki, 4.3 km SSW of Tongoa, NW flank of Mt Nokilalaki, ca. 500 m SSE of Shelter 2, tree-inventory plot Nokilalaki 1, 1°14.7'S, 120°09.2'E, 1900 m, Aug 2007: *Culmsee 2636* (sterile; CEB, L!) & *2641* (sterile; BO [BO-1938462]!, CEB, GOET [GOET020007]!) & *2721* (sterile; CEB, K [K000993487]!).

Kab. Tojo Una-una, Kec. Ulubongka. N slope of Mt Katopas, 1°9.8'S, 121°26.9'E, 1100 m, 4 Sep 2014: Sight record by F Brambach (photograph Figure 1f).

**West Sulawesi (Sulawesi Barat)**: Kab. Mamasa. Kec. Mamasa, near Osango c. 2°56'S, 119°19'E (“Celebes en Ond. Boven Binoeang, ca. Osango”), c. 1500 m, 1 Jul 1939: *Netherland's Indies Forest Service (NIFS) bb 28293* (sterile; L [L.2529832]!).

**South Sulawesi (Sulawesi Selatan)**: Kab. Luwu, Kec. Ponrang, near Kampung Tampa, c. 3°11'S, 120°13'E (“Celebes en Ond. Palopo, Bakka, Kampoeng Tampa”), c. 100 m, 15 Sep 1941: *NIFS bb 33081* (flowers; L [L.2535805]!).

Kab. Luwu Timur: Kec. Malili, Ussu, c. 2°36'S, 121°06'E (“Selebes, Malili, Oesoe): c. 300 m, 13 Jul 1931: *NIFS Cel./II-385* (flower buds; L [L.2535679]!); ibid. loco, c. 400 m, 19 Jun 1934: *NIFS Cel./II-293* (sterile; L [L.2517541]!); ibid. loco, 100 m, 28 Mar 1941: *NIFS bb 32595* (sterile; BO [BO-1304600], L [L.2517463]!).

Kab. Luwu Timur, Kec. Wasuponda, Larona, c. 2°45'S, 121°20'E, 500–1000 m (“Celebes. Goud. Celebes, Ond. afd. Malili, nabij La Rona”), n.d.: *NIFS bb 1843* (sterile; L [L.2535842]!) & *bb 1895* (sterile; L [L.2535843]!).

Kab. Luwu Timur, Kec. Nuha, Hills W of Soroako, c. 2°31'S, 121°19'E, 550 m, 17 Jun 1979: *van Balgooy MMJ 3767* (old inflorescences; L [L.2535910]! [wood sample L 0708626]).

**Figure 2.**
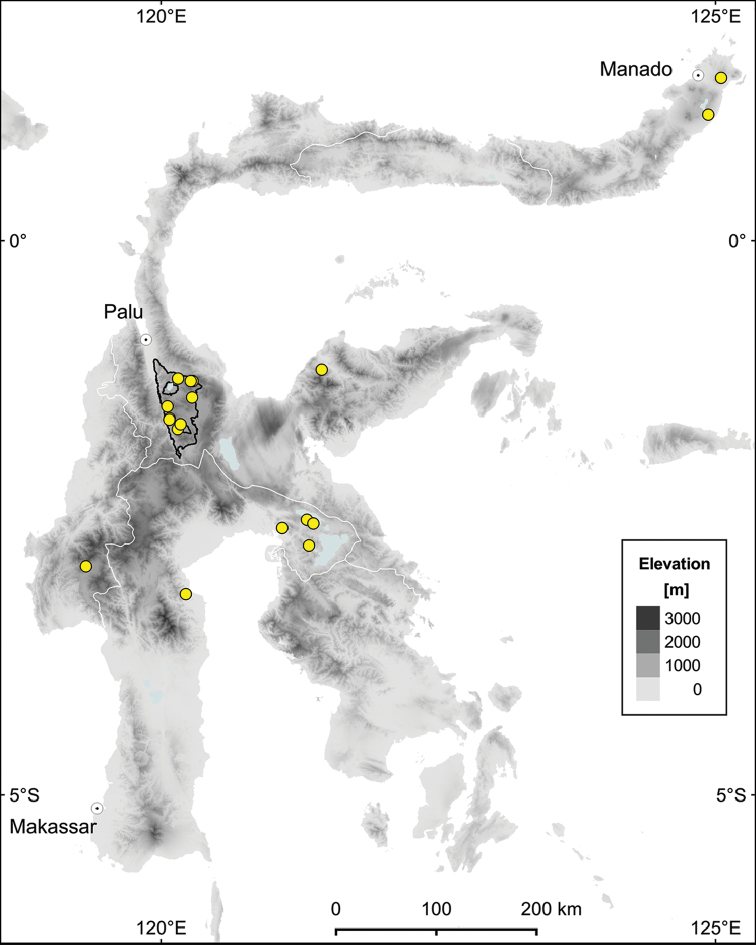
Distribution map of *Syzygium balgooyi* in Sulawesi. Collecting localities are shown as yellow dots; Lore Lindu National Park is indicated by a black line. Map created with QGIS (QGIS Development Team 2016) using the digital elevation model of Jarvis et al. (2008).

### 2. Syzygium contiguum

Taxon classificationPlantaeMyrtalesMyrtaceae

Brambach, Byng & Culmsee sp. nov.

urn:lsid:ipni.org:names:60474722-2

Figures 3 , 4 , 8

Myrtaceae “ sp. 9” (Culmsee and Pitopang 2009).

#### Diagnosis.

*Syzygium contiguum* is a species of treelets with slender, angular young branchlets and (sub-)sessile, chartaceous leaves with few (8–13), distinct secondary veins, two marginal veins, and conspicuous cordate bases; the basal lobes of opposed leaves often reach each other. The dense or lax paniculate inflorescences are terminal or arise from the upper leaf axils and bear small (5–6 × 3–4 mm in mature buds) pyriform flowers with numerous white stamens. The species is similar to *Syzygium urdanetense* (Elmer) Merrill (1951, 420) from the Philippines but differs from that species by angular (vs usually terete) young branchlets and inflorescence axes, by smaller (usually 9–14 × 3.5–5 vs 18–35 × 6–11cm), chartaceous (vs coriaceous) leaves with shorter (0–1.5 vs 3–5 mm) petioles and fewer secondary vein pairs (8–13 vs 17–35), and by gland-dotted (vs smooth) petals. It differs from *Syzygium paucipunctatum* (Koord. and Valeton) Merrill and Perry (1939, 169) from Sumatra, Java, and Borneo, in chartaceous (vs coriaceous), leaves with no or few gland dots (vs gland-dotted beneath) which dry dark reddish brown to very dusky red above and (dark) reddish brown beneath (vs. olive-green above and brownish beneath) and shorter (5–6 vs c. 9 mm long) mature flower buds. Floral formula B1 Bt2 K4* C4* A∞* Ĝ(2)┼ Vx~8.

#### Type.

INDONESIA. Central Sulawesi (Sulawesi Tengah), LLNP, Kab. Sigi, Kec. Kulawi, 2.4 km ENE of Toro, NW of Pono Valley, tree-inventory plot Pono, 1°29.7'S, 120°03.4'E, 1050 m, Jul 2006: *Culmsee H 535* (flowers; holotype L[L.3962133]!, isotype CEB).

#### Description.

**Treelets**, up to 10 m tall, diameter at breast height ≤ 11 cm. **Bark** and wood not known. Young **branchlets** 0.5–1 × 1–2 mm, slender, rectangular in cross section, sometimes narrowly winged, epidermis drying dark reddish brown, smooth; soon becoming terete with 4 ridges and eventually terete, bark pale or yellowish brown with flaking remnants of epidermis; with (1–) 2 (–4) pairs of ≤ 2 mm long, caducous cataphylls near the base of the current flush.

**Leaves** opposite, (sub-)sessile. Petioles 0–1.5 × 1–2 mm, absent or very short and stout, drying very dusky red. Blades (6.5–) 9–14 (–19) × (2.3–) 3.5–5 (–6.1) cm, ratio (1.9–) 2.5–3.2 (–3.6), narrowly elliptic or lanceolate, rarely oblanceolate, base distinctly cordate (or auriculate), basal lobes of opposed leaves often touching each other, apex (long-)acuminate or caudate, margin flat or sometimes minutely revolute; chartaceous, drying dull to satin, dark reddish brown to very dusky red above, (dark) reddish brown beneath; sometimes with scattered black gland dots. Midrib channelled above, prominent, rounded, and darker than the lamina beneath. Secondary vein pairs 8–12, (3–) 5–11 (–18) mm apart, slightly sunken or sometimes slightly prominent, rather inconspicuous above, very prominent and darker than the lamina beneath; some intersecondary veins usually present. Tertiary veins sub-parallel near the midrib to reticulate towards the margin, faint above, prominulous beneath. Inner intramarginal vein 3–7 mm from leaf margin, hardly looping; outer intramarginal vein 0.5–2 mm from leaf margin.

**Inflorescences** terminal and in the axils of 1–2 distal leaf-pairs, ± lax panicles, (2.5–) 3.5–7.5 (–11) cm long, peduncles 1–3.5 cm long, axes (sub-)angular, flattened. Bracts c. 0.5–2 (–7) mm long, lowermost foliaceous, caducous, others deltate, keeled, ± persistent; bracteoles 2 per flower, 0.5–1 mm long, similar to bracts.

**Flowers** ≤ 40 per inflorescence, within the panicles in monads or triads, 4-merous, anthopodium absent, c. 15 mm in diameter at anthesis, mature buds 5–6 × 3–4 mm. Hypanthium 4–5 × 3–5.5 mm, obconical to infundibuliform, gland-dotted or ± smooth, hypanthium rim 2 mm long. Calyx lobes 0.5–1 × 1–2.5 mm, deltate first, becoming broadly rounded and eventually splitting irregularly at anthesis. Petals 3–6 × 3–6 mm, pseudocalyptrate, orbicular, gland-dotted. Stamens c. 80–100, filaments 6–10 mm long, white, anthers c. 0.5 mm long, ellipsoid. Ovary bilocular, locules subtended by spongy tissue, ovules c. 8 per locule, spreading. Style 6–8 mm long, pointed.

**Fruits** 2-seeded, 1.1–1.3 × 1.8–1.9 cm, globose to oblate, drying smooth, pericarp c. 2 mm thick, hypanthium rim c. 5 mm in diameter.

**Seeds** 9–10 × 12–13 mm, half-moon shaped.

#### Etymology.

The specific epithet refers to the leaf bases of opposing leaves which, due to their cordate shape and the short petioles, often approach or touch each other.

#### Phenology.

In Central Sulawesi a slight dry season usually lasts from May to September or October. Flowering was observed during the wet and dry seasons: in July 2016, January/February 2007, July 2007 in Pono and in April 1975 on Mt Nokilalaki.

#### Distribution and habitat.

According to our present knowledge, the species is endemic to the province of Central Sulawesi. It has been recorded from only three localities in and around LLNP at 1000–1150 m elevation (Figure 4). Most of the specimens were collected in our (FB and HC) inventory plot in Pono Valley near the western border of LLNP.

In the Pono inventory plot, the species was found in undisturbed submontane rainforest on flat terraces with Sideralic Cambisols (IUSS Working Group WRB 2014) developed from metamorphic rocks. The forest at Pono was dominated by Fagaceae, Lauraceae, Sapotaceae, Moraceae, and Rubiaceae species (families with top five FIV) and contained seven other species of *Syzygium*: *S. acuminatissimum* (Blume) de Candolle (1828, 261), *S. balgooyi*, *S. galanthum*, *S. lineatum* (DC.) Merrill and Perry (1938a, 109), *S. phaeostictum* Merrill and Perry (1942, 270), and two undetermined species (Brambach et al. in press). See Culmsee and Pitopang (2009) for more information on the floristics of the Pono valley plot. The collection locality of *Widjaja EAW 3502* in the almost entirely deforested Napu valley suggests remnant riparian forest as habitat.

#### Conservation status.

*Syzygium contiguum* has a limited geographical distribution (estimated EOO 557 km²) and seems to be restricted to submontane forest within a narrow elevational belt. We assume that the estimated AOO of 12 km² is unrealistically low, due to limited collection activities in Central Sulawesi. However, only the collection locality of *Meijer 9572* seems to be covered by intact forest habitat. The other two localities are small forest fragments (*Widjaja EAW 3502*) and forest with recent deforestation activities in close proximity (Pono inventory plots, detected using the Global Forest Change website, Hansen et al. 2013), possibly related to the establishment of cocoa plantations (Aiyen Tjoa, Tadulako University, personal communication, June 2015). Given the apparent narrow geographical and elevational distribution and the recommendation to use a precautionary attitude in conservation assessments (IUCN Standards and Petitions Subcommittee 2014) we propose a preliminary extinction risk assessment of “Endangered” (EN B1ab(i,ii,iii)).

#### Notes.

*Syzygium urdanetense* (as *Eugenia urdanetensis*, Elmer 1914, 2356), the species most similar to *S. contiguum*, was originally described from Mt Masay (previously Mt Urdaneta) on the southern Philippine island of Mindanao and is widespread throughout the Philippines (Merrill 1951, Pelser et al. 2011). The species is variable in vegetative characters such as leaf size, leaf base (usually rounded and only the very base cordate, but sometimes distinctly cordate) and branchlet shape (usually terete, but rarely subangular). In addition to the characters mentioned in the diagnosis, there are differences in the tertiary venation, the veins being ± ladder-like and perpendicular to the midrib in *S. urdanetense* whereas in *S. contiguum* they are ± parallel to the secondary veins near the midrib and become reticulate towards the leaf margin (Figure 8f). While with the available material, *S. contiguum* can be clearly distinguished from *S. urdanetense* on morphological grounds, we do not discard the possibility that future collections, especially from the northern peninsula of Sulawesi, will uncover populations with intermediate characters. If so, *S. contiguum* may eventually have to be sunk into an expanded *S. urdanetense*. In light of the almost complete lack of taxonomic resolution for *Syzygium* in Sulawesi, we nevertheless consider it advisable to propose *S. contiguum* as a distinct species.

Two fruiting specimens collected at low elevations (200–300 m) on Sulawesi’s Southeast Peninsula, *Prawiroatmodjo & Maskuri 1231* [L.2517450] and *1957* [L.2517547], are morphologically similar to *S. contiguum* as defined above except for the leaf tips which are not long-acuminate. In the absence of flowering material, and because of the different habitat and distribution, we prefer not to include them here at present, but future additional collections may prove otherwise.

We choose *Culmsee 535* as type specimen because it contains flowers in all stages of maturity although unfortunately, it was collected with only two duplicates (in CEB and L). Nevertheless, the more widely distributed paratypes collected by HC at the type locality all belong to the same population as the type.

#### Additional specimens examined

(Paratypes). INDONESIA. **Central Sulawesi (Sulawesi Tengah), LLNP**: Kab. Poso, Kec. Nokilalaki, N slopes of Mt Nokilalaki. (“Celebes, central part, area of Mt. Nokilalaki, Loro Kalimata Reserve”), 1°13'S, 120°08'E, ± 1000 m, 24 Apr 1975: *Meijer 9572* (flowers; L [L.2535817]!, US [US-2995269] photo!).

Kab. Poso, Kec. Lore Peore, Road to Napu from camp Dongi-dongi, 1°31.2'S, 120°22.4'E, 1127 m, 26 Dec 1988: *Widjaja EA EAW 3502* (fruits; BO [BO-1917489]! [BO-1917490]!, K! , L).

Kab. Sigi, Kec. Kulawi, 2.4 km ENE of Toro, NW of Pono Valley, tree-inventory plot Pono, 1°29.7'S, 120°03.4'E, 1050 m, Jul 2006: *Culmsee H 284* (flowers; GOET [GOET020010]!) .

Kab. Sigi, Kec. Kulawi, 2.4 km ENE of Toro, NW of Pono Valley, tree-inventory plot “Pono”, 1°29.7'S, 120°03.4'E, 1050 m, Jan 2007: *Culmsee H y410* (flower buds; BO [BO-1938450]! [BO-1938451]!, CEB, GOET [GOET020012]!, K [K000993488]!, L!); ibid. loco, Jul 2007: *Culmsee H r463* (flower buds; BO [BO-1938464]!, CEB, GOET [GOET020011]!, K [K000993489]!, L!!) & *y503* (flower buds; CEB, GOET [GOET020013]!) & *y514* (flower buds; BO [BO-1938452]!, CEB) & *y581* (flower buds; CEB, L!) & *y582* (flower buds; CEB, K [K000993490]!) & *y592* (flower buds; BO [BO-1938453]! [BO-1938454]!, CEB) & *y595* (flower buds; CEB, GOET [GOET020014]!, L!).

**Figure 3.**
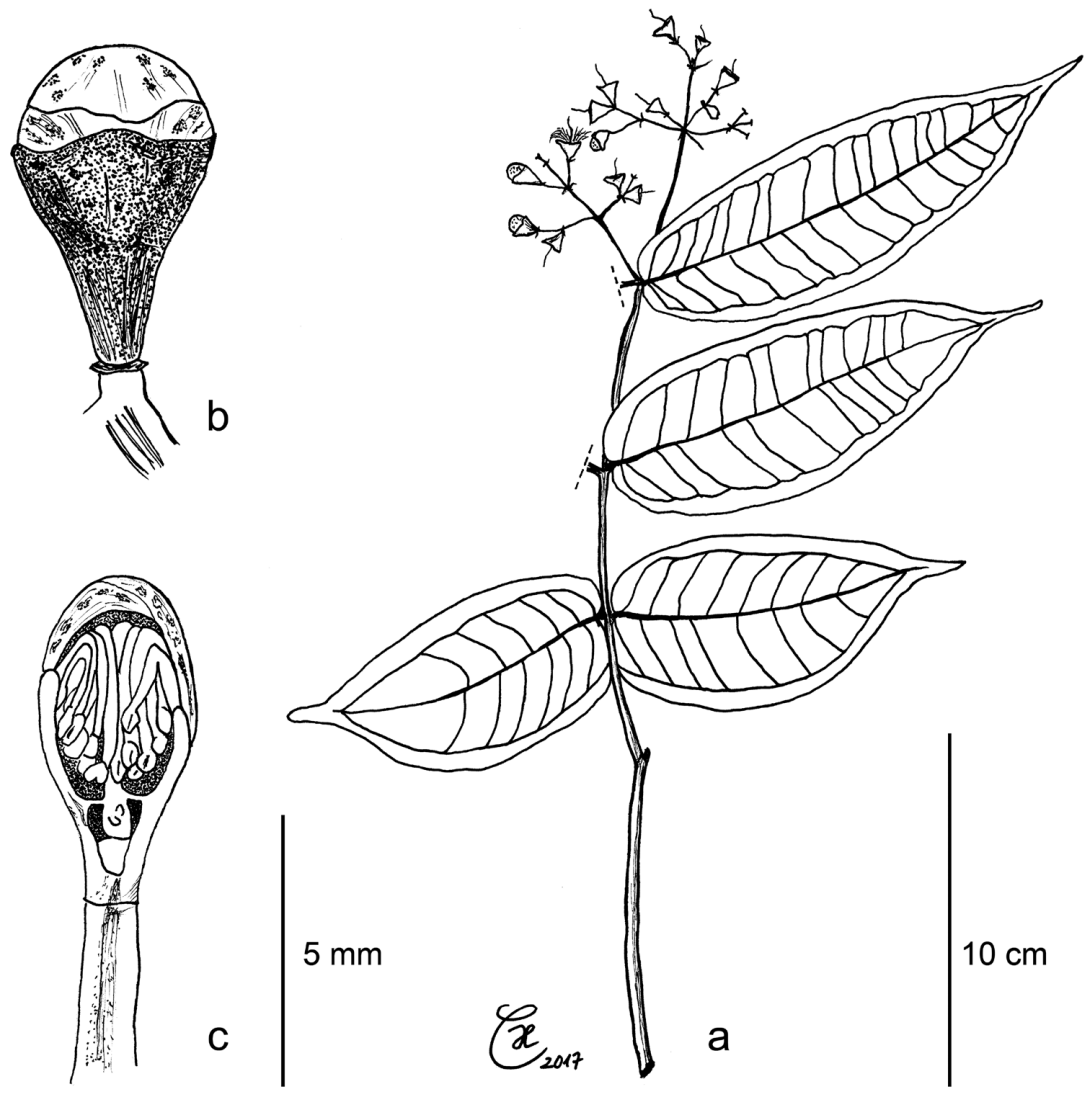
*Syzygium contiguum*: **a** leafy twig with flowers in different developmental stages **b** flower bud with gland-dotted petals and shallow calyx lobes **c** flower bud, longitudinal section. All from holotype *Culmsee 535*.

### 3. Syzygium devogelii

Taxon classificationPlantaeMyrtalesMyrtaceae

Brambach, Byng & Culmsee sp. nov.

urn:lsid:ipni.org:names:60474723-2

Figures 4 , 5 , 8

Myrtaceae „ sp. 10“ p.p. (Culmsee and Pitopang 2009, see also 2017 (Erratum)).

#### Diagnosis.

*Syzygium devogelii* is a species of treelets characterised by slender, narrowly winged young branchlets, medium-sized narrowly elliptic leaves, straight and distinct secondary veins connected by an intramarginal vein impressed above and prominent beneath, small flowers (5 × 3 mm in bud) in terminal inflorescences that develop into rather large fruits (c. 20 × 25 mm), mature seeds lacking a testa, and cotyledons with echinate outer surfaces. The species is morphologically similar to *Syzygium perspicuinervium* (Merr.) Masamune (1942, 537) but differs from that species in smaller leaves with fewer secondary veins and in flowers with distinct calyx lobes (vs calyx calyptrate). It is furthermore similar to *Syzygium valdevenosum* (Duthie) Merrill and Perry (1939, 182) but differs in lateral veins which are impressed above (vs prominent), much smaller inflorescences, and smaller, obconical (vs infundibuliform) flowers. Floral formula B1 Bt2 K4* C4* A∞* Ĝ(2)┼ Vx∞.

#### Type.

INDONESIA. Central Sulawesi (Sulawesi Tengah), LLNP, Kab. Poso, Kec. Lore Utara, west slope of Mt Rorekautimbu, c. 1°17.5'S 120°16.3'E, 1350 m, 11 May 1979: *de Vogel EF 5293* (fruits; holotype L [L.2535665]! [L.2535666]!; isotype K!).

#### Description.

**Trees**, up to 13 m tall, diameter at breast height ≤ 13 cm, trunk ≤ 7 m tall. Outer **bark** whitish to brown, mealy or peeling off in thin sheets, inner bark pale or dark red, wood cream-coloured. Young **branchlets** 1–2.5 × 2–3 mm, ± flattened, angular or oblong in cross section with 4 narrow wings, epidermis dark red when young, drying reddish or yellowish brown, smooth; becoming rounded with 4 ridges, bark (yellowish) brown, peeling off in thin sheets.

**Leaves** (sub-)opposite. Petioles 7–16 × 1–3 mm, channelled above, rounded beneath, epidermis drying smooth or with transverse cracks. Blades (12.5–) 14–19 (–22.5) × (4–) 4.5–7 (–8.5) cm, ratio (2.1–) 2.6–3.3 (–4), narrowly elliptic (or lanceolate), base cuneate or obtuse, apex acuminate, margin revolute; chartaceous or coriaceous, red or pink when young, above, beneath, drying dull to satin, variable in colour from greyish brown and olive grey to very dusky red above, dull to satin and, dark reddish brown beneath; pellucid dots rather few, visible or not on both sides. Midrib channelled above, very prominent, rounded, smooth and drying darker than the lamina beneath. Secondary vein pairs (9–) 11–14 (–17), 5–22 mm apart, channelled or impressed above, prominent and drying darker than the lamina beneath, straight or slightly arching from the midrib; intersecondary veins sometimes present. Tertiary veins dense, ± ladder-like and perpendicular to the midrib, faint above, prominulous beneath. Inner intramarginal vein 2–9 mm from leaf margin, looping or not and prominent; outer intramarginal vein 0.5–1.5 mm from leaf margin, as prominent as tertiary venation.

**Inflorescences** terminal, dense metabotryoids, 2.5 cm long, peduncles 1 cm long, axes flattened, with 2 or 4 narrow wings, drying brown. Bracts c. 1.5 mm long, ovate, keeled, caducous; bracteoles 2 per flower, 1 mm long, similar to bracts.

**Flowers** c. 15 per inflorescence, within the inflorescence in triads, 4-merous, anthopodium absent, only known before anthesis, mature buds 5 × 3 mm. Hypanthium c. 4 × 3 mm, obconical, drying dark reddish brown, densely glandular-warty, hypanthium rim 2 mm long, glandular inside. Calyx lobes c. 1 × 2 mm, broadly rounded. Petals c. 3 × 3 mm, cucullate in bud. Stamens c. 100, filaments 2–3 mm long, anthers c. 0.4 mm long, ellipsoid. Ovary bilocular, surrounded by spongy tissue, ovules numerous per locule, ascending. Style 3–4 mm long, pointed.

**Fruits** 1-seeded, c. 20 × 25 mm, irregularly depressed globose, laterally compressed, green, drying black and, smooth, pericarp ± woody, 1 mm thick, hypanthium rim 1–2 mm long, 5–9 mm in diameter.

**Seeds** c. 15 × 20 mm, transverse ellipsoid, testa adhering to the pericarp, spongy inside and adhering to the outer surface of the cotyledons, cotyledons ± half-globose, facing surfaces undulate, outer surfaces densely echinate, protuberances obscured by spongy testa tissue.

#### Etymology.

The species is named after Eduard Ferdinand de Vogel (*1942). Ed de Vogel is a renowned authority on Malesian orchids, especially those from New Guinea. His contributions to the flora of Sulawesi are perhaps less well known: with almost 2000 specimens of excellent quality collected there in 1973–74 and 1979 – among them the type specimen of this species – he was one of the most prolific plant collectors on the island during the 20^th^ century.

#### Phenology.

Flowering was recorded in August, fruiting in May.

#### Distribution and habitat.

**Figure 4.**
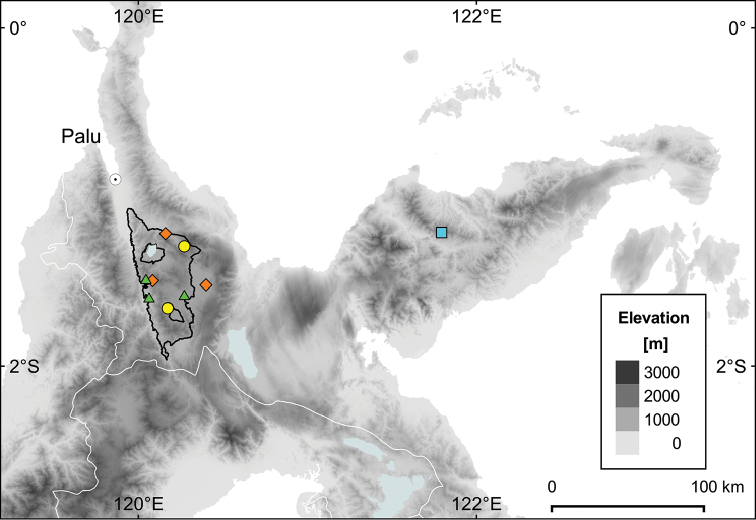
Distribution map of four species of *Syzygium* in Central Sulawesi: *Syzygium contiguum* (orange diamonds), *S. devogelii* (yellow dots), *S. eymae* (light blue square), and *S. galanthum* (green triangles). Lore Lindu National Park is indicated by a black line. Map created with QGIS (QGIS Development Team 2016) using the digital elevation model of Jarvis et al. (2008).

*Syzygium devogelii* is endemic to the province of Central Sulawesi, currently known to occur in lower montane forest at two localities in LLNP from 1350–1400 m elevation (Figure 4). In the Bariri NE inventory plot, it was fairly common, growing on mid-slope terraces with Rhodic Ferralsols (IUSS Working Group WRB 2014) derived from acid plutonic rocks. The forest there was dominated by Fagaceae, Myrtaceae, Burseraceae, Lauraceae, and Elaeocarpaceae (families with top five FIV) and contained six other species of *Syzygium*: *S. acuminatissimum*, S. aff. baeuerlenii (F.Muell.) Craven and Biffin (in Craven et al. 2006, 135), *S. lineatum*, *S. zeylanicum* (L.) de Candolle (1828, 260), and two undetermined species (Brambach et al. in press). See Culmsee and Pitopang (2009) for more information on the floristics of the Bariri forest.

#### Conservation status.

*Syzygium devogelii* has a limited geographical distribution and seems to be restricted to lower montane forest within a narrow elevational belt. Known from only two localities, the EOO and AOO cannot be estimated reliably for the species. Because of the low collection density in Central Sulawesi, we believe that the species is more widespread and common than it currently appears. Deforestation has been recorded close to the type locality (using the Global Forest Change website, Hansen et al. 2013). Given the apparent narrow geographical and elevational distribution, ongoing deforestation and the recommendation to use a precautionary attitude in conservation assessments (IUCN Standards and Petitions Subcommittee 2014) we propose a preliminary extinction risk assessment of “Endangered” (EN B1ab(i,ii,iii)).

#### Notes.

**Figure 5.**
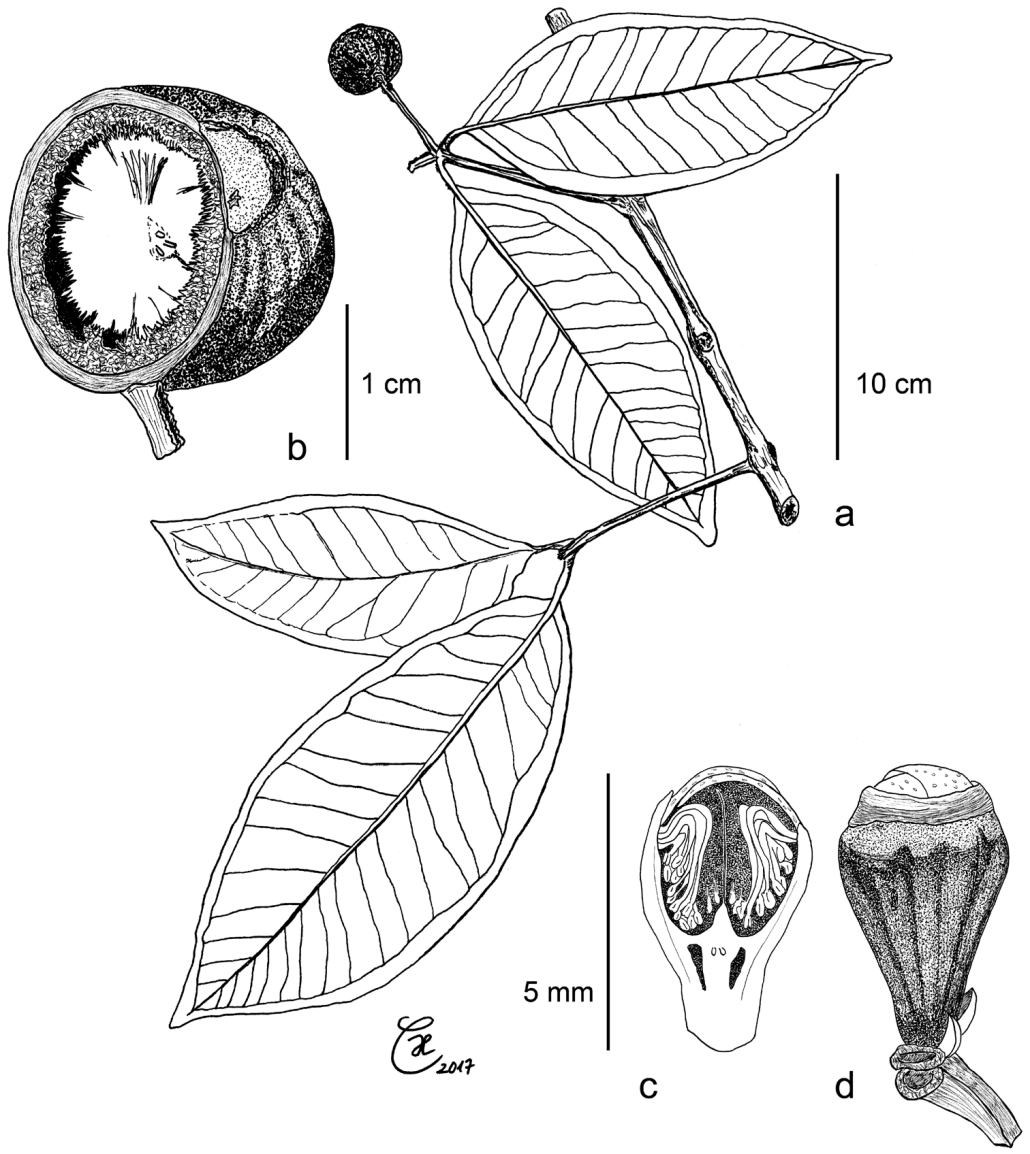
*Syzygium devogelii*: **a** leafy twig with fruit **b** longitudinal section of ripe fruit with inner, flat side of cotyledon and echinate outer surface **c** flower bud, longitudinal section **d** flower bud, exterior view. **a–b** holotype de *Vogel 5293* **c–d** *Culmsee 1564*.

Most species of *Syzygium* are reported to have cotyledons with rather smooth outer surfaces, unlike the peculiar echinate cotyledons of *S. devogelii*. We here interpret the tissue covering the outer surface of the cotyledons (Figure 5) and obscuring its protuberances as derived from the testa, as reported for the Australian species *Syzygium bungadinnia* (F.M.Bailey) Hyland (1983, 64), but closer examinations of fruit and seed structures are necessary to corroborate this interpretation.

Juvenile specimens of *Syzygium balgooyi* are similar to *S. devogelii* in their leaf shape, colour, and venation. In fact, both species were treated as one morphotype in Culmsee and Pitopang (2009, 2017). Besides the very different flowers, they can, however, be distinguished by the shape of the young branchlets: strongly flattened and with rounded ridges in *S. balgooyi* (Figure 1e) vs ± flattened with 4 narrow wings in *S. devogelii* (Figure 5).

#### Additional specimens examined

**(Paratypes). INDONESIA. Central Sulawesi (Sulawesi Tengah), LLNP**, Kab. Poso, Kec. Lore Tengah: 9 km NW of Bariri, 100 m east of climate tower, tree-inventory plot Bariri NE, 1°39.4'S, 120°10.5'E, 1400 m, 9 Sep 2006: *Culmsee H 1333* (sterile; BO [BO-1938455]!, CEB) & *1378* (sterile; CEB, K [K000993491]!); ibid. loco, 18 Aug 2011: *Brambach F, Mangopo H, Firdaus, Faber M, Tiranda R 0818* (sterile; BO [BO-1938442]!, CEB, GOET [GOET020015]!) & *0845* (sterile; BO [BO-1938443]!, CEB, L!).

9 km NW of Bariri, 80 m south of climate tower, tree-inventory plot Bariri S, 1°39.5'S, 120°10.4'E, 1400 m, Jul 2006: *Culmsee H 1252* (sterile; CEB, GOET [GOET020016]!) & *1564* (flower buds; CEB, L!).

### 4. Syzygium eymae

Taxon classificationPlantaeMyrtalesMyrtaceae

Brambach, Byng & Culmsee sp. nov.

urn:lsid:ipni.org:names:60474724-2

Figures 4 , 6 , 8

#### Diagnosis.

*Syzygium eymae* is characterised by small (usually 2.3–4 × 1.5–2.5 cm), (sub-)sessile, leaves with thickly coriaceous, (broadly) elliptic or obovate blades, dense terminal inflorescences, and small, pyriform flowers with a calyptrate calyx that bears a minute apical opening and splits irregularly at anthesis. It differs from the morphologically similar *Syzygium paradoxum* (Merr.) Masamune (1942, 536) in angular young branchlets (vs terete), leaves without conspicuous gland dots (vs leaves conspicuously gland-dotted beneath), fewer pairs of secondary veins (5–7 vs 10–14), and smaller (5–6 × 3 vs c. 12 × 5 mm in mature buds), pyriform flowers without anthopodia (vs infundibuliform with anthopodia 5–7 mm long). Floral formula B1 Bt2 (K4?* C4?*) A∞* Ĝ(2)┼ Vx?.

#### Type.

INDONESIA. Central Sulawesi (Sulawesi Tengah), Kab. Tojo Una-Una, Border of Kec. Ulubongka and Kec. Ampana Tete, Mt Lumut, between summit and western secondary peak, c. 1°12.3'S, 121°47.6'E, ± 2200 m (“Selebes, Res. Menado. O.afd. Poso. G. Lóemoet, Pilaartop en W. bijtop. (summit)”), 5 Sep 1938: *Eyma 3624* (flowers; holotype U [U.1439024]!; isotypes: BO [BO-1679767]!, L [L.2535689]!).

#### Description.

**Trees**, height, **bark** and wood unknown. Young **branchlets** slender, 0.5–1 × 1–2 mm, rectangular in cross section, ridges arising at the petioles and running downwards to next node, epidermis drying reddish black; remaining angular or becoming ± terete, bark reddish brown and scaly; with 1–2 pairs of minute cataphylls near the base of the current flush.

**Leaves** opposite, (sub-)sessile. Petioles 0.5–2 × 1–1.5 mm, absent or very short and stout, drying black. Blades (1.8–) 2.3–4 (–5.2) × (1.2–) 1.5–2.5 (–3.1) cm, ratio 1.3–2, (broadly) elliptic or (broadly) obovate, base obtuse or rounded, apex rounded, acute, or shortly acuminate, margin revolute; thickly coriaceous (c. 0.3 mm thick), dull, drying dark reddish grey to reddish black above, (very) dusky red beneath; without black gland dots. Midrib impressed above, prominent, rounded, and darker than the lamina beneath. Secondary vein pairs (4–) 5–7, 3–10 mm apart, channelled and inconspicuous above, (slightly) prominent and more reddish than the lamina beneath; intersecondary veins sometimes present. Tertiary veins reticulate, channelled above, indistinct beneath. Inner intramarginal vein 1–2 mm from leaf margin, looping; outer intramarginal vein not present.

**Inflorescences** terminal, 2-nodate metabotryoids, ≤ 3 cm long, peduncles ≤ 1 cm long, axes angular. Bracts c. 1–1.5 mm long, deltate, keeled, caducous; bracteoles 2 per flower, c. 1 mm long, similar to bracts.

**Flowers** ≤ 10 per inflorescence, within the inflorescence in triads, anthopodium absent, mature buds 5–6 × 3 mm. Hypanthium 4–5 × 4–5 mm, pyriform, smooth, hypanthium rim 1.5 mm long. Calyx lobes calyptrate with small apical opening, slightly lighter-coloured than hypanthium when dry, splitting irregularly at anthesis, caducous. Petals calyptrate, adhering to the calyx. Stamens c. 50, filaments 6–7 mm long, white, anthers c. 0.4 mm long, ellipsoid. Ovary bilocular, ovules several per locule, ascending. Style 6–7 mm long, pointed.

**Fruits** and **seeds** unknown.

#### Etymology.

The species is named after Pierre Joseph Eyma (1903-1945), one of the early botanists to explore the mountainous regions of Central Sulawesi (Eyma 1940, van Steenis-Kruseman and van Welzen 2014). Eyma collected many valuable specimens from high-elevation areas, including the type specimen of this species.

#### Phenology.

The species was collected in flowering state in September 1938.

#### Distribution and habitat.

*S. eymae* is endemic to the province of Central Sulawesi and currently only known from the type locality: Mt Lumut on Sulawesi`s eastern peninsula (Figure 4). No information on habitat is given on the label of the type specimen. Mt Lumut is made up of ultramafic rocks (Geological Research and Development Centre 1993) and upper montane (cloud) forest would be the expected vegetation type there at 2200 m.

#### Conservation status.

With only the type specimen known, we consider *S. eymae* “Data Deficient” (DD) at present, following the IUCN Red List Categories and Criteria (IUCN 2012).

#### Notes.

The species of tribe *Syzygieae* Wilson (in Wilson et al. 2005, 15) bearing a calyptrate calyx have mostly been treated under the genus *Cleistocalyx* Blume (1850, 84, see Merrill and Perry 1937). The calyptrate calyx is a relatively rare character, currently known to occur in only about 30 of the > 1200 species of *Syzygieae* (Merrill and Perry 1937, Chantaranothai and Parnell 1993, Takeuchi 2002, Biffin et al. 2005, Craven and Biffin 2010). Its occurrence, however, is widely spread over the phylogenetic tree of the tribe; so *Cleistocalyx* is not monophyletic and has therefore been synonymised under an expanded *Syzygium* (Craven et al. 2006, Craven and Biffin 2010).

**Figure 6.**
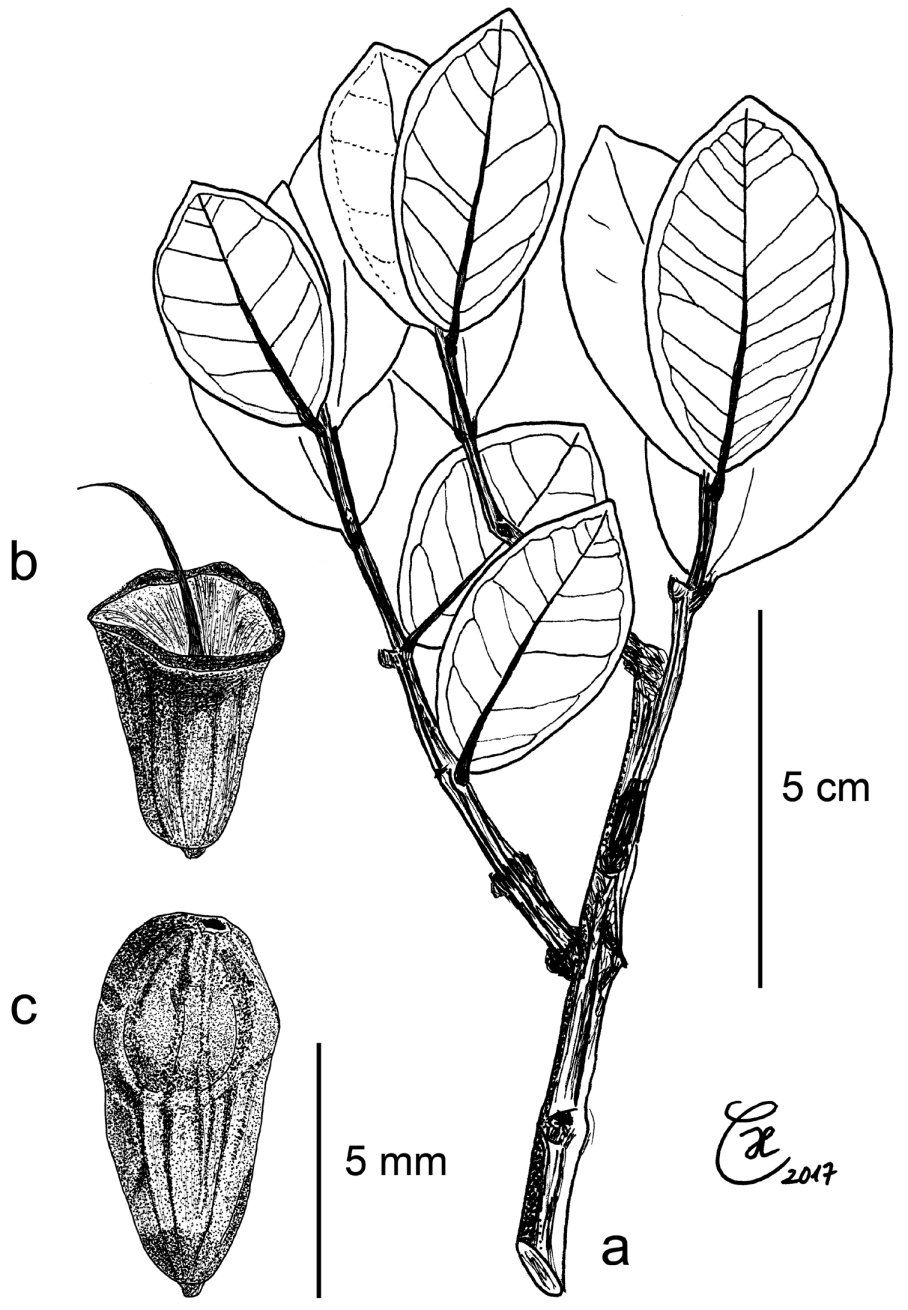
*Syzygium eymae*: **a** leafy twig **b** flower after shedding of calyx and stamens **c** closed pyriform flower, calyptrate calyx with minute apical opening. All drawings from isotype (L) *Eyma 3624*.

The flowers of *Cleistocalyx* are described as having “calyptrate calyces, the undivided, often more or less indurated upper parts of which fall as a lid”, the lid often remaining attached at one side of the flower at early anthesis (Merrill and Perry 1937). In *S. eymae*, the calyx clearly has the form of a calyptra, but at anthesis it splits irregularly into four or five parts, starting with a minute (< 0.5 mm diam.) apical opening (Figure 6). One or several of the irregular segments may remain attached to the hypanthium rim shortly after anthesis before eventually being shed. The mode of dehiscence of the calyx thus seems to represent an intermediate condition between *Cleistocalyx* and classical *Syzygium*, similar to the situation in *Syzygium apodophyllum* (F.Muell.) Hyland (1983, 49) from Queensland, Australia.

Most species of *Syzygium* with calyptrate calyces are clearly different from *S. eymae* in their much larger leaves with more pairs of secondary veins. The few small-leaved species can all be easily distinguished: *S. paradoxum* from Borneo differs by the characters given in the diagnosis. *S. pseudocalcicola* Craven & Biffin (in Craven et al. 2006, 139) from the Philippines and *S. canicortex* Hyland (1983, 66) from Queensland have many, closely parallel secondary veins and caudate leaf apices, *S. apodophyllum* has ovate leaves with a long-acuminate apex and clavate flowers.

*Syzygium eymae* is also superficially similar *S. paucivenium* (Merr.) Merrill (1951, 408) from Taiwan and the Philippines, but can easily be distinguished from that species by the leaves with channeled, inconspicuous secondary veins on the upper surface (vs. prominent and distinct), smaller inflorescences (<10 vs 20–30 flowers), smaller flowers (mature buds 5–6 vs. 9 mm long), and the presence of the calyptrate calyx (vs. truncate to shallowly lobed).

Several specimens collected on Mt Rorekautimbu in LLNP at 2400 m (e.g. *Brambach et al. 0768*) may belong here. They are morphologically similar to the type specimen, but have longer petioles. Since we currently lack flowering material of these specimens and because of the large distance between the respective collection localities, we prefer to await more specimens before incorporating these collections in *S. eymae*.

### 5. Syzygium galanthum

Taxon classificationPlantaeMyrtalesMyrtaceae

Brambach, Byng & Culmsee sp. nov.

urn:lsid:ipni.org:names:60474725-2

Figures 4 , 7 , 8

Myrtaceae “ sp. 7” (Culmsee and Pitopang 2009)

#### Diagnosis.

*Syzygium galanthum* is similar to *Syzygium hylochare* (Diels) Merrill and Perry (1942, 249) from New Guinea but differs from that species in larger leaves (usually 15–22 vs 8–14 cm long), more slender flowers with longer anthopodia (5–10 vs 3–5 mm) and milky white petals (vs pink or red). It is also similar to the widely cultivated *Syzygium malaccense* (L.) Merrill & Perry (1938b, 215) but has subangular (vs clearly angular) and more slender branchlets (2–3 vs 6–8 mm in diameter), smaller, chartaceous leaves (vs coriaceous), more slender inflorescences, more slender flowers with longer anthopodia (5–10 mm vs 0–5 mm), hypanthia which dry reddish brown with many black glands (vs drying dark brown without conspicuous glands), and creamy-white petals (vs pink or red). Floral formula B1 Bt2 K2:2┼ C4* A∞* Ĝ(2)┼ Vx∞.

#### Type.

INDONESIA. Central Sulawesi (Sulawesi Tengah): LLNP, Kab. Poso, Kec. Lore Tengah, 3.5 km NE of Rompo, following road to Katu for 3 km, then following footpath N for 2 km, tree-inventory plot Tarara, 1°35.3'S 120°17.0'E, 1200 m, 29 Nov 2011: *Brambach F, Mangopo H, Firdaus, Faber M, Tiranda R 1316* (flowers; holotype L[L.3962132]!; isotypes BO [BO-1938381]!, CEB, GOET [GOET020017]!, K [K000993484]!).

#### Description.

**Trees**, up to 25 m tall, diameter at breast height ≤ 30 cm, trunk straight, ≤ 15 m tall, with buttresses 0.4 m tall, sometimes with stilt roots. Outer **bark** bright- or rusty red, peeling off in thin sheets, inner bark pale or dark red, wood straw or cream-coloured. Young **branchlets** 1–2 × 2–3 mm, subangular, flattened, epidermis olive, drying reddish brown, striate; becoming ± terete, bark (reddish) brown, striate or fissured, later peeling off in small thin sheets.

**Leaves** (sub-)opposite. Petioles 6–18 × 1–3 mm, channelled above, rounded beneath, turning corky, pale brown, drying (reddish) brown. Blades (10–) 12–23 (–26) × (4–) 5.5–7.5 (–9) cm, ratio (1.7–) 2.5–3.2 (–3.5), (narrowly) elliptic or rarely oblanceolate, base acute, obtuse, or rounded, apex acuminate, acumen often recurved, margin flat or revolute; chartaceous, glossy green and often ± bullate above, paler green beneath, drying dull and (greyish or olive) brown above, dull and (yellowish or greyish) brown beneath; pellucid dots scattered or numerous, usually visible on lower surface and sometimes also on upper surface. Midrib channelled above, prominent, rounded, drying pale or reddish brown, striate and with dark gland dots beneath. Secondary vein pairs (6–) 8–10 (–12), 7–25 mm apart, prominulous or not, concolorous with the lamina and usually inconspicuous above, prominent and concolorous with or more reddish than the lamina beneath; intersecondary veins present. Tertiary veins reticulate, lax, prominulous or not, concolorous with the lamina and usually inconspicuous above, prominent and concolorous with or more reddish than the lamina beneath. Inner intramarginal vein 3–8 mm from leaf margin, (strongly) looping; outer intramarginal vein 1–3 mm from leaf margin.

**Inflorescences** axillary on leafless portion of the twigs, often fascicled, lax, (sub-)sessile botryoids or monads, 3–5 cm long, peduncles absent or ≤ 1 cm long, axes angular, drying (reddish) brown with many black gland dots, turning corky at the base, often with conspicuous whitish blisters. Bracts c. 0.5 mm long, early caducous; bracteoles 2 per flower, similar to bracts.

**Flowers** 1–8 per inflorescence, within the inflorescence in monads, 4-merous, only known before anthesis, mature buds 15–25 × 5–7 mm, anthopodium 5–10 (–14) mm long, slender. Hypanthium 7–11 × 5–7 mm, infundibuliform, pale green, drying dark reddish brown, wrinkled, densely black gland-dotted and with conspicuous whitish blisters, hypanthium rim 3 mm long. Calyx lobes 2 × 3–5 (outer) and 3–4 × 5–7 (inner) mm, broadly rounded with thin hyaline margins, greenish white, drying red, sparsely gland-dotted. Petals c. 8 × 6 mm, hood shaped before anthesis, milky white, drying yellowish red, faintly veined and densely pellucid-dotted. Stamens c. 100, filaments 4–10 mm long before anthesis, yellowish green, anthers c. 1 mm long, ovoid or ellipsoid, yellow. Ovary bilocular, locules surrounded by spongy tissue, ovules many per locule, ascending. Style 10 mm long before anthesis, pointed, green.

**Fruits** and **seeds** unknown.

#### Etymology.

**Figure 7.**
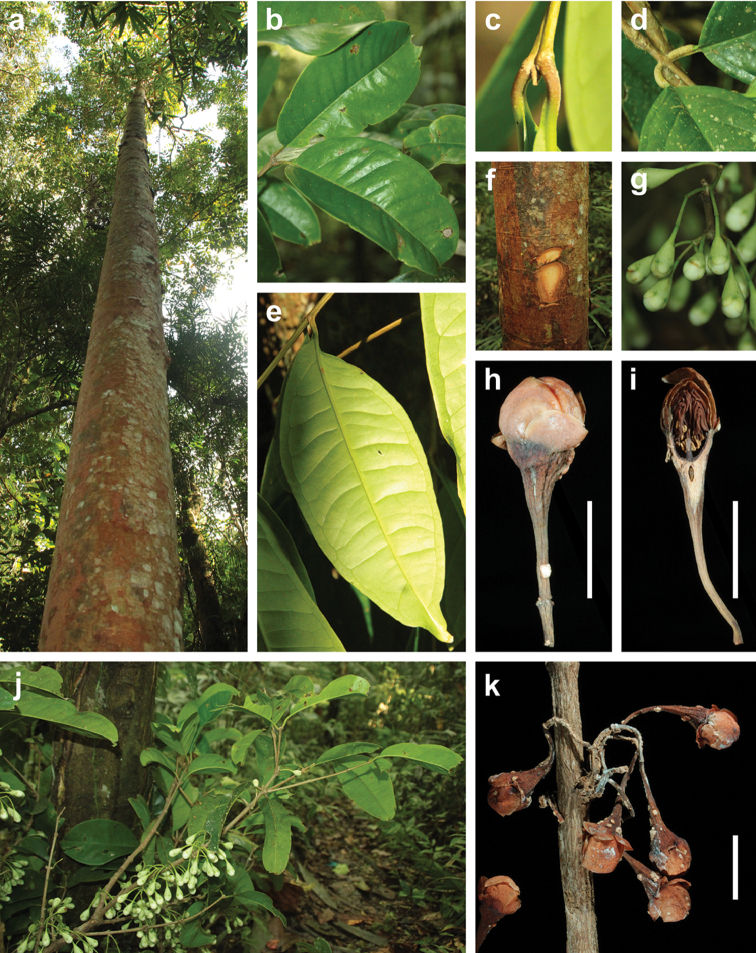
Morphological characters of *Syzygium galanthum*. **a** trunk, c. 15 m tall **b** upper side of leaves **c** branchlet tip with smooth younger petioles **d** older corky petioles **e** underside of leaf **f** bark slash **g** mature flower buds in fresh state **h** dried mature flower bud with apical part of inflorescence axis and white blister on the anthopodium **i** longitudinal section of mature flower bud in dried state **j** branch with mature flower buds below the leaves **k** detail of dried, fascicled inflorescences. **a–b** and **f–k** type collection *Brambach et al. 1316* **c** and **e** *Brambach et al. 1083* **d** *Brambach et al. 1047*. All scale bars: 1 cm.

The species name derives from the Greek *γάλα* (milk) and *άνθος* (flower) and refers to the petals’ milky white colour (Figure 7g, j). The colour pattern of the flowers is furthermore similar to the one found in the amaryllidaceous genus *Galanthus* Linnaeus (1753, 288).

#### Phenology.

The type specimen was collected with mature flower buds in late November, suggesting flowering in December.

#### Distribution and habitat.

*Syzygium galanthum* is currently only recorded from LLNP in the province of Central Sulawesi (Figure 4). There it occurs scattered in undisturbed submontane forest at three localities from 700–1200 m over Sideralic Cambisols and mollic Umbrisols derived from varied parent material. The forests at these localities were dominated by species of Fagaceae, Lauraceae, Moraceae, and Sapotaceae, among others.

#### Conservation status.

*Syzygium galanthum* has a limited geographical distribution (estimated EOO 140 km²) and seems to be restricted to submontane forest between 700 and 1200 m. We assume that the estimated AOO of 12 km² is unrealistically low, due to limited collection activities in Central Sulawesi. However, despite being inside the protected LLNP, recent deforestation activities have been detected near one of the collection sites (Pono inventory plot, detected using the Global Forest Change website, Hansen et al. 2013), possibly related to the establishment of cocoa plantations (Aiyen Tjoa, Tadulako University, personal communication June 2015). Given the ongoing deforestation activities in the species’ narrow geographical range and the recommendation to use a precautionary attitude in conservation assessments (IUCN Standards and Petitions Subcommittee 2014) we propose a preliminary extinction risk assessment of “Endangered” (EN B1ab(i,ii,iii)).

#### Vernacular name.

Tambeanitu (Bahasa Behoa, *Brambach et al. 1316*).

#### Notes.

In the field, *S. galanthum* can be recognised by the leaves with corky petioles and rather few, ± arching secondary veins. Similar corky petioles occur in *S. peregrinum* (Blume) Merrill & Perry (1939, 154) from Borneo and the Southern Philippines. A peculiarity is the presence of white blisters on the inflorescence axes and flowers of dried material (Figure 7h, k). These blisters were not observed in fresh state and must have appeared during the drying process.

It appears that there is a group of morphologically similar species in Malesia, all characterised by pale-drying leaves with rather few secondary veins, inflorescences below the leaves, and medium-sized to large, showy, infundibuliform flowers with short or long anthopodia and either white or red/pink petals and stamens: e.g. *S. iliasii* Ashton (2011, 222) from Borneo, *S. galanthum* and several unnamed collections from Sulawesi, *S. hylochare*, *S. laqueatum* Merrill & Perry (1942, 257), and *S. phaeostictum* from New Guinea and possibly the Maluku Islands, and the widely cultivated *S. malaccense* with unknown geographical origin. As can be seen from material in L, the assignation of specimens to these species has not been consistent in the past and specific limits in the group need to be critically revised.

#### Additional specimens examined

**(Paratypes). INDONESIA. Central Sulawesi (Sulawesi Tengah), LLNP**: Kab. Poso, Kec. Lore Tengah, 3.5 km NE of Rompo, following road to Katu for 3 km, then following footpath N for 2 km, tree-inventory plot Tarara, 1°35.3'S 120°17.0'E, 1200 m, 22 Nov 2011: *Brambach F, Mangopo H, Firdaus, Faber M, Tiranda R 1047* (sterile; BO [BO-1938446]!, CEB, GOET [GOET020018]!) & *1083* (sterile; BO [BO-1938445]!,CEB, L!) & *1290* (sterile; BO [BO-1938444]!, CEB, K [K000993485]!).

Kab. Sigi, Kec. Kulawi, 2.4 km ENE of Toro, NE edge of Pono Valley, tree-inventory plot Pono, 1°29.7'S, 120°03.4'E, 1050 m, 16 Aug 2006: *Culmsee 537* (sterile; CEB, K [K000993492]!) & *890* (sterile; BO [BO-1938448]!, CEB); ibid. loco, Jul 2007: *Culmsee r497* (sterile; BO [BO-1938449]!, CEB, GOET [GOET020019]! , L!).

Kab. Sigi, Kec. Kulawi Selatan, 4 km ENE of Watukilo, 400 m N of Mboe River, tree-inventory plot Rantena, 1°36.2'S, 120°04.5'E, 700 m, 17–26 Jun 2011: *Brambach F, Mangopo H, Firdaus, Faber M, Tiranda R 0533* (sterile; BO [BO-1938447]!, CEB, L!).

**Figure 8.**
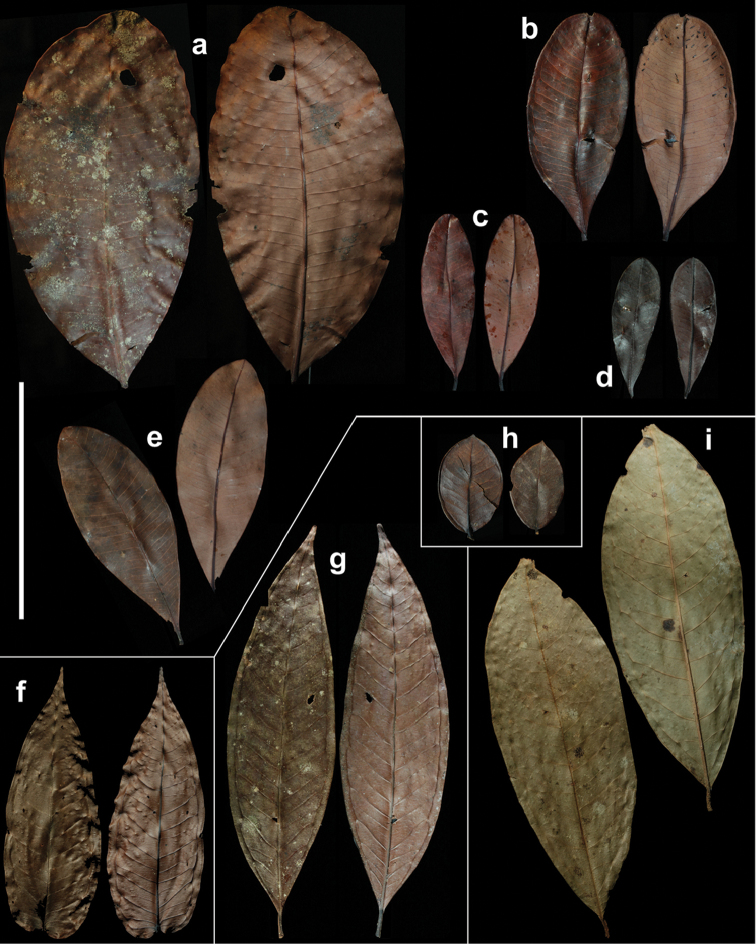
Leaves of all new species described. Variation of *Syzygium balgooyi* (**a–e**), *S. contiguum* (**f**), *S. devogelii* (**g**), *S. eymae* (**h**), and *S. galanthum* (**i**). **a** *Brambach et al. 0283* **b** *Brambach et al. 0681* **c** *Brambach et al. 1333* **d** *de Vogel 5413* [L.2517563] **e** *Culmsee r2162* **f** *Culmsee r463* **g** *Brambach et al. 0818* **h** *Eyma 3624* [L.2535689] **i** *Brambach et al. 0533*. Scale bar: 10 cm, valid for all leaves.

## Supplementary Material

XML Treatment for Syzygium balgooyi

XML Treatment for Syzygium contiguum

XML Treatment for Syzygium devogelii

XML Treatment for Syzygium eymae

XML Treatment for Syzygium galanthum
